# Clinical significance of the increased expression of the WT1 gene in peripheral blood of patients with acquired aplastic anemia

**DOI:** 10.1002/jha2.563

**Published:** 2022-09-20

**Authors:** Ken Ishiyama, Tran Cao Dung, Tatsuya Imi, Kohei Hosokawa, Yasuhito Nannya, Hirohito Yamazaki, Seishi Ogawa, Shinji Nakao

**Affiliations:** ^1^ Department of Hematology Kanazawa University Hospital Kanazawa Ishikawa Japan; ^2^ Department of Pathology and Tumor Biology Graduate School of Medicine Kyoto University Kyoto Japan; ^3^ Division of Hematopoietic Disease Control Institute of Medical Science The University of Tokyo Tokyo Japan; ^4^ Institute for the Advanced Study of Human Biology Kyoto University Kyoto Japan; ^5^ Center for Hematology and Regenerative Medicine Karolinska Institute Stockholm Sweden

**Keywords:** aplastic anemia, myelodysplastic syndrome, *WT1* mRNA copy number

## Abstract

To determine the significance of increased *Wilms tumor 1* (WT1) gene expression in the peripheral blood of patients with acquired aplastic anemia (AA), we analyzed serial changes in *WT1* mRNA copy number (WT1cn) in 63 patients with AA as well as in five patients with myelodysplastic syndromes (MDS) and seven patients with paroxysmal nocturnal hemoglobinuria (PNH). WT1cn was higher than the cut‐off (≥50 copies/μg RNA) at the time of the first measurement in 41% of untreated (60–190 copies/μg RNA [median 130]) and 59% of treated (59–520 copies/μg RNA [median 150]) AA patients. Although WT1cns gradually increased in most AA patients during the 2–105 months follow‐up period, they did not lead to clonal evolution except in three patients in whom the maximum change ratio of WT1cn (WT1cn‐change max), defined as the ratio of WT1cn at the first examination to that of the maximum value, exceeded 20.0 and who developed MDS at 2, 46, and 105 months. Increased WT1 gene expression was enriched in granulocytes rather than in mononuclear cells in most WT1‐positive AA patients and did not correlate with mutations of genes associated with myeloid malignancy. WT1cns were high at 690–5700 (median 2000) in MDS patients and remained high thereafter, while WT1cns in PNH patients (77–200; median 96) were similar to those in AA. Thus, moderate increases in WT1cns up to 600 are common in AA patients in stable remission. An increase in the WT1cn‐change max over 20.0 may portend transformation from AA to MDS.

## 1 INTRODUCTION

*Wilms tumor 1* (*WT1*), a tumor suppressor gene, is overexpressed in immature blasts from patients with hematological malignancies and other tumors [1, 2, 3]. Since the number of copies of *WT1* mRNA (WT1cn) correlates well with the burden of acute myeloid leukemia (AML), WT1cn has been used as a reliable marker of residual AML cells [4, 5, 6]. Previous studies have shown that the WT1cn/μg peripheral blood (PB) RNA determined using a quantitative polymerase chain reaction (PCR) method is less than 50 in healthy individuals (HIs) and in patients with AML in deep remission [7]. An increase in WT1cn to more than 200 copies/μg RNA in PB predicts the relapse of AML in patients in hematological remission after chemotherapy [8] or allogeneic hematopoietic stem cell transplantation (allo‐HSCT) [9]. Measurement of WT1cn has also been shown to be useful in distinguishing high‐risk myelodysplastic syndrome (MDS) from low‐risk MDS as well as diagnosing disease progression to more advanced MDS with time in patients with low‐risk MDS [10].

Acquired aplastic anemia (AA) is a syndrome characterized by pancytopenia and bone marrow (BM) hypoplasia, caused primarily by the T‐cell attack on hematopoietic stem cells (HSCs) [11]. Although immunosuppressive therapy (IST) induces hematologic remission in approximately 70% of patients with severe AA (SAA) and produces long‐term survival in 90% of these patients, 10%–15% of them develop secondary MDS/AML up to 5–10 years after treatment [12]. Recent studies have shown that somatic mutations in genes related to myeloid malignancy are detectable in approximately 30% of AA patients at the time of diagnosis [13, 14], suggesting a potential risk of MDS/AML development in these patients. Because the prognosis of secondary myeloid malignancy is extremely poor unless allo‐HSCT is performed early in the disease [15], it is important to predict the evolution to MDS in AA patients after treatment; however, no useful markers have been established to detect such evolution.

Some studies have shown that WT1cn is very low or undetectable in patients with AA at diagnosis and have suggested the potential usefulness of measuring WT1cn in distinguishing AA from MDS [16, 17]. However, the clinical significance of measuring WT1cn in patients with AA remains unclear because previous studies examined PB only in a small number of patients with AA at diagnosis and did not assess the change in WT1cn after treatment. Serial examination of WT1cns in PB may be useful in detecting early signs of clonal evolution in patients with AA.

To address this question, we examined 63 patients with AA for WT1cn in PB and assessed the correlation of the change in WT1cn with their clinical outcomes and mutations of myeloid malignacy‐related genes.

## 2 PATIENTS AND METHODS

### 2.1 Patients

A total of 75 patients with BM failure (63 with AA, five with MDS, and seven with paroxysmal nocturnal hemoglobinuria [PNH]) treated at Kanazawa University Hospital between February 2014 and March 2021 were enrolled in this study. The treatments that 17 untreated AA received after the first sampling are provided in Supporting Information S1. This study was approved by the Institutional Review Board (#2468), and all participants gave their informed written consent to participate in this study.

### 2.2 WT1cn measurement, detection of glycosylphosphatidylinositol‐anchored protein‐deficient (GPI[‐]) cells in PB, and deep sequencing of myeloid malignancy‐related genes

PB samples from the patients were tested for WT1cn using a quantitative PCR kit (Otsuka Pharmaceutical Co., Tokyo, Japan) [7]. WT1cn of PB was expressed as copies/μg RNA, and the cut‐off value of the assay between HIs and patients was defined as 50 copies/μg RNA. WT1cn was measured serially over time at an interval of 1–45 months. A BM examination was performed when a patient's blood cell counts decreased without apparent cause. GPI(‐) blood cells were detected using high‐sensitivity flow cytometry [18]. Deep sequencing was performed to search for gene abnormalities associated with myeloid malignancies in 32 patients with an increase in WT1cn as described in Supporting Information S2 [14, 19]. The method for a droplet digital PCR (ddPCR) assay to measure WT1cn in granulocytes and the formula to calculate the WT1cn in granulocytes are shown in Supporting Information S3 and S4. The method for a qualitative PCR assay to assess the WT1 gene expression in granulocytes and statistical analysis are provided in Supporting Information S5 and S6, respectively.

## 3 RESULTS

### 3.1 WT1cns at the time of initial measurement in the untreated AA patients

Of the 17 untreated patients (13 non‐severe and four severe) shown in Table 1, 10 (59%) were negative (<50 copies/μg RNA) for WT1 expression while seven had increased WT1cns ranging from 60 to 190 copies (median 130) at the time of initial measurement. There were no significant differences between the 10 WT1‐negative and seven WT1‐positive patients in terms of clinical parameters such as gender, age, severity, dependence on blood transfusions, presence of GPI(‐) cells, laboratory data at the first WT1cn measurement, and time from onset to initial WT1cn measurement (Table 1). The initial WT1cns of two patients who later converted to MDS (AA27) and PNH (AA28) were <50 and 60, respectively.

**TABLE 1:** Characteristics of untreated aplastic anemia (AA) patients who were positive or negative for increased *WT1* mRNA copy numbers (WT1cns)

	WT1cn ≥50 copies/μg RNA			
	Total	Positive	Negative	*p*‐Value
Number of patients	17	7	10	
Gender				
Female	9 (52.9%)	3 (42.9%)	6 (60.0%)	0.64
Male	8 (47.1%)	4 (57.1%)	4 (40.0%)	
Age, median (range)	55 (29–88)	59 (33–88)	55 (29–74)	0.63
AA severity				
Non‐severe	13 (76.5%)	5 (71.4%)	8 (80.0%)	1.0
Severe	4 (23.5%)	2 (28.6%)	2 (20.0%)	
Transfusion dependency				
Yes	5 (29.4%)	3 (42.9%)	2 (20.0%)	0.59
No	12 (70.6%)	4 (57.1%)	8 (80.0%)	
GPI(‐) cells				
Yes	7 (41.2%)	3 (42.9%)	4 (40.0%)	1.0
No	10 (58.8%)	4 (57.1%)	6 (60.0%)	
Blood cell count at the initial WT1cn measurement, median (range)				
Neutrophil (×10^9^/L)	1.3 (0.06–3.0)	1.1 (0.35–2.5)	1.4 (0.06–3.0)	0.70
Reticulocyte (×10^9^/L)	44 (9–88)	40 (16–74)	50 (9–88)	0.26
Platelet (×10^9^/L)	25 (5–64)	24 (6–32)	28 (5–64)	0.56
Time from diagnosis to the initial WT1cn measurement (month), median (range)	2 (0–293)	3 (0–293)	2 (0–136)	0.84
WT1cn at the first measurement, median (range)^a^	<50 (<50 to 190)	130 (60–190)	<50 (<50 to <50)	<0.001

Abbreviations: AA, aplastic anemia; GPI(‐) cells, glycosylphosphatidylinositol‐anchored protein‐deficient cells.

^a^ WT1cns of negative cases (<50 copies/μg RNA) were arbitrarily set as 30 copies/μg RNA to calculate the median value of WT1cn.

### 3.2 Changes in the WT1cns over time

**FIGURE 1:**
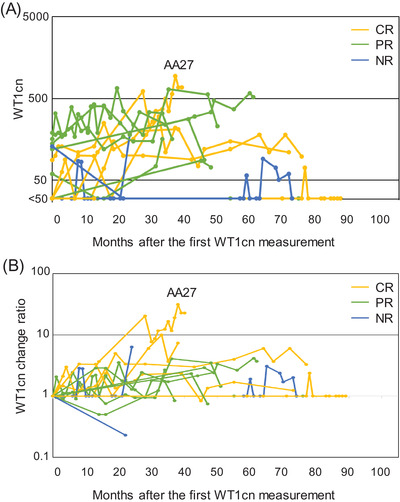
Changes in the *WT1* mRNA copy number (WT1cn) in the peripheral blood in patients with untreated aplastic anemia (AA). (A) Changes in the WT1cn over time (*n* = 16). The yellow (*n* = 6), green (*n* = 7), and blue (*n* = 3) lines denote the patients who achieved complete response (CR), partial response (PR), and no response (NR) to treatment. (B) Changes in the WT1cn change ratio calculated by dividing WT1cn at a given time point by WT1cn at the time of initial testing over time. Only a patient (AA27) in whom the maximum change ratio of WT1cn (WT1cn‐change max) defined as the ratio of WT1cn at the first examination to that of the maximum value exceeded 20.0 developed myelodysplastic syndrome (MDS) at 28 months.

The WT1cns of the 16 patients, except one patient in whom serial measurements were not performed (AA59), varied considerably, as shown in Figure 1A. Seven of the 10 patients with negative results at the first WT1cn measurement became positive (≥50 copies/μg RNA) for WT1cn during the follow‐up period of 3–78 months (median 16 months); five patients remained positive until the last measurement while two patients were transiently positive during the observation period. The WT1cn change ratio, calculated by dividing WT1cn at a given time point by WT1cn at the time of initial testing, also varied during the observation period and tended to be higher in patients with complete response or partial response than in nonresponders, although the difference was not significant (Figure 1B). During the median 48 months (range 13–89 months) of observation, WT1cn change ratios ranged from 1.0 to 20.3 (median 3.3). WT1cn‐change max was 10.0 or less in 15 patients (94%), whereas the ratio was 31.3 in one patient (AA27, 6%) who eventually progressed to MDS.

### 3.3 WT1cns at the time of initial measurement in previously treated patients with BM failure

To further clarify the clinical significance of the WT1cn increase in AA patients, we analyzed the results of WT1cn measurement in 58 patients (46 with previously treated AA including two patients transformed to MDS later [five with severe and 41 with non‐severe AA at the time of sampling], five with MDS with multilineage dysplasia [MLD], all of whom had been transformed from AA, and seven with hemolytic PNH secondary to AA). Characteristics of the treated cohort are shown in Table 2. A total of six patients (two with AA, one with AA who transformed to MDS, and three with MDS) underwent allo‐HSCT later during the study period.

**TABLE 2:** Characteristics of the treated patients with bone marrow failure

	Total	AA	MDS	PNH
Number of patients	58	46	5	7
Gender				
Female	28 (48.3%)	24 (52.2%)	0 (0.0%)	4 (57.1%)
Male	30 (51.7%)	22 (47.8%)	5 (100.0%)	3 (42.9%)
Age, median (range)	58 (22–85)	55.5 (22–85)	71 (57–81)	60 (54–72)
AA severity				
Severe		5 (10.9%)		
Non‐severe		41 (89.1%)		
Transfusion dependency				
Yes	13 (22.4%)	10 (21.7%)	3 (60.0%)	0 (0.0%)
No	45 (77.6%)	36 (78.3%)	2 (40.0%)	7 (100.0%)
GPI(‐) cells				
Yes	21 (36.2%)	12 (26.1%)	2 (40.0%)	7 (100.0%)
No	36 (62.1%)	33 (71.7%)	3 (60.0%)	0 (0.0%)
Not tested	1 (1.7%)	1 (2.2%)	0 (0.0%)	0 (0.0%)
Blood cell count at the initial WT1cn measurement, median (range)				
Neutrophil (×10^9^/L)	1.3 (0.2–6.9)	1.3 (0.3–3.6)	1.3 (0.2–6.9)	1.6 (0.8–4.2)
Reticulocyte (×10^9^/L)	52 (7–400)	48 (7–90)	29 (8–63)	17 (91–400)
Platelet (×10^9^/L)	38 (6–21)	30 (6–21)	63 (24–152)	115 (63–200)
Time from diagnosis to the initial WT1cn measurement (month), median (range)	114 (7–457)	114 (7–457)	58 (14–426)	256 (81–447)
WT1cn at the first measurement, median (range)^a^	91.5 (<50 to 5700)	72 (<50 to 520)	2000 (690–5700)	96 (77–200)

Abbreviations: AA, aplastic anemia; GPI(‐) cells, glycosylphosphatidylinositol‐anchored protein‐deficient cells; MDS, myelodysplastic syndromes; PNH, paroxysmal nocturnal hemoglobinuria.

^a^ *WT1* mRNA copy numbers (WT1cns) of negative cases (<50 copies/μg RNA) were arbitrarily set as 30 copies/μg RNA to calculate the median value of WT1cn.

The median WT1cn at the first measurement in 46 treated AA patients was 72 (range <50 to 520). The median WT1cn of 10 transfusion‐dependent AA patients (80 [<50 to 310]) was comparable to that of 36 transfusion‐independent AA patients (66 [<50 to 520]). The initial WT1cns of two AA patients who were later transformed to MDS were <50 and 330, respectively. Nineteen (41%) were negative, while 27 showed increased WT1cns, ranging from 59 to 520 copies (median 150) at the time of the first measurement. The 27 patients with increased WT1cns had a disease duration of 91 months (range 7–457) that was comparable to that in 19 patients negative for WT1cns (median 127 months; range 14–434). There were no significant differences between the WT1cn‐positive group and negative group according to gender, age, AA severity (non‐severe vs. severe), dependence on blood transfusion, the presence of GPI(‐) cells, laboratory data (neutrophil count, reticulocyte count, and platelet count) at the first WT1cn measurement, and time from onset to the initial WT1cn measurement (Table 3).

**TABLE 3:** Characteristics of the treated aplastic anemia (AA) patients who were positive or negative for increased *WT1* mRNA copy numbers (WT1cns)

	WT1cn ≥50 copies/μg RNA			
	Total	Positive	Negative	*p*‐Value
Number of patients	46	27	19	
Gender				
Female	24 (52.2%)	13 (48.1%)	11 (57.9%)	0.56
Male	22 (47.8%)	14 (51.9%)	8 (42.1%)	
Age, median (range)	56 (22–85)	55 (22–85)	63 (26–78)	0.61
AA severity				
Non‐severe	41 (81.9%)	24 (88.9%)	17 (89.5%)	1.0
Severe	5 (10.9%)	3 (11.1%)	2 (10.5%)	
No. of transfusion‐dependent cases	10 (21.7%)	6 (22.2%)	4 (21.1%)	1.0
GPI(‐) cells				
Positive	12 (26.1%)	8 (29.6%)	4 (21.1%)	0.48
Negative	33 (71.7%)	19 (70.4%)	14 (73.7%)	
Blood cell count at the initial WT1cn measurement, median (range)				
Neutrophil (×10^9^/L)	1.3 (0.32–3.6)	1.1 (0.32–3.6)	1.5 (0.45–2.7)	0.29
Reticulocyte (×10^9^/L)	48 (7–87)	49 (16–87)	43 (7–78)	0.17
Platelet (×10^9^/L)	30 (6–213)	30 (7–198)	29 (6–213)	0.65
Time from diagnosis to the initial WT1cn measurement (month), median (range)	114 (7–457)	91 (7–457)	127 (14–434)	0.30
WT1cn at the first measurement, median (range)^a^	72 (<50 to 520)	<50 (<50 to <50)	150 (59–520)	<0.001

Abbreviations: AA, aplastic anemia; GPI(‐) cells, glycosylphosphatidylinositol‐anchored protein‐deficient cells.

^a^ WT1cns of negative cases (<50 copies/μg RNA) were arbitrarily set as 30 copies/μg RNA to calculate the median value of WT1cn.

The median value of WT1cn at the time of the first measurement was 2000 (690–5700) in five MDS patients and 96 (77–200) in seven PNH patients.

### 3.4 Changes in the WT1cns in the different groups of patients

**FIGURE 2:**
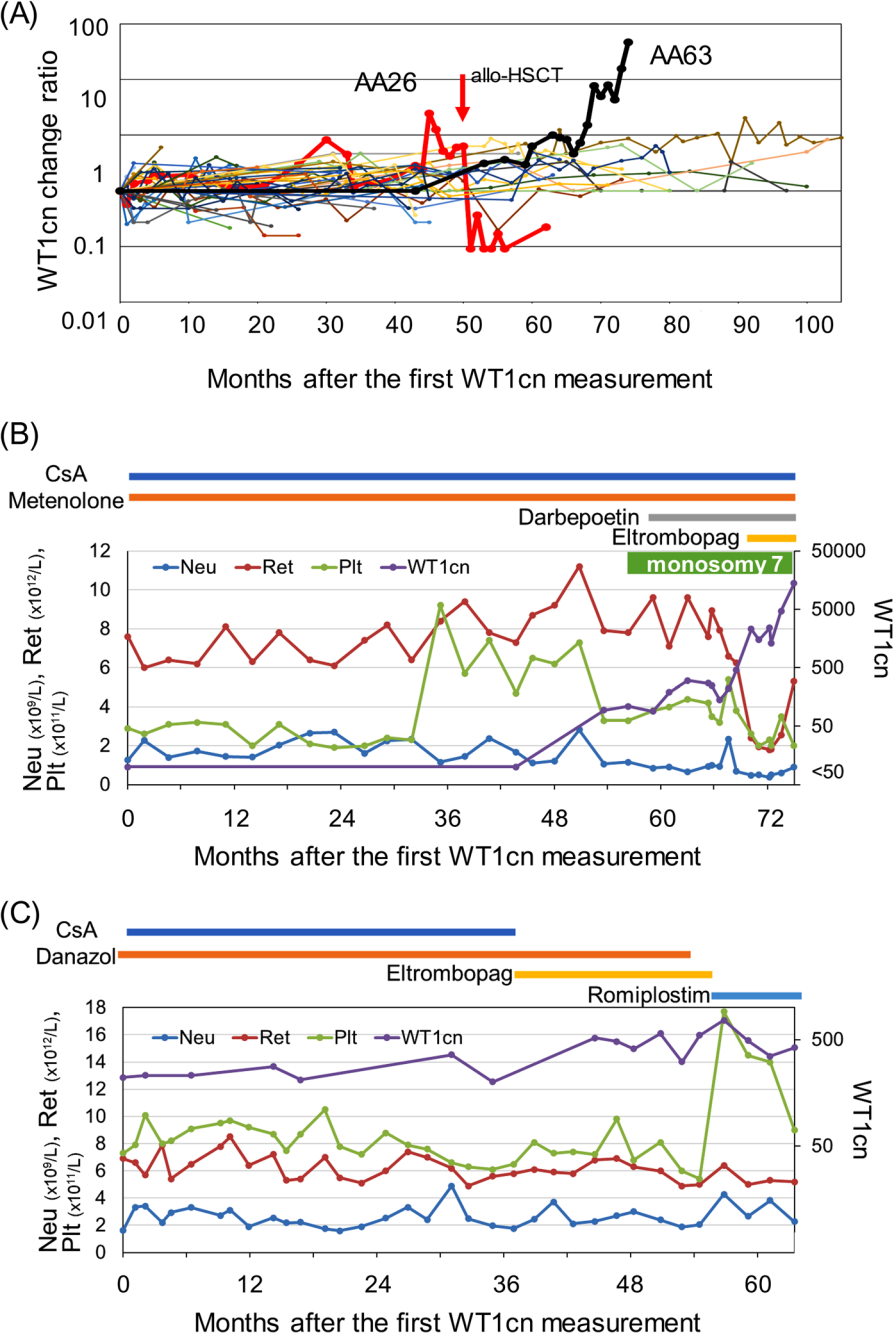
Changes in the *WT1* mRNA copy numbers (WT1cns) in the peripheral blood (PB) in the treated aplastic anemia (AA) patients The WT1cn change ratios of individual patients are shown in different colors. (A) Patients with treated AA (*n* = 46). The red (AA26) and black bold lines (AA63) indicate the transitions of two patients who eventually progressed to myelodysplastic syndromes (MDS) during the observation period. The red arrow indicates the time point of allogeneic hematopoietic stem cell transplantation (allo‐HSCT). (B) Clinical courses of an AA patient (AA63) with high WT1cns (range <50 to 14,000 copies; WT1cn‐change max, 466.7) who developed monosomy 7. (C) Clinical course of an AA patient (AA31) showing a persistent increase in the WT1cns (range 200–760 copies; WT1cn‐change max, 3.5) without any signs of progression to MDS. Abbreviations: CsA, cyclosporine; Neu, neutrophil; Plt, platelet; Ret, reticulocyte

Figure 2 illustrates the WT1cn in AA patients. WT1cn change ratios in treated AA patients ranged from 1.0 to 466.7 (median 3.4) during a median observation period of 70 months (range 3–112 months). The WT1cn‐change max was less than 10.0 in 42 patients (91%), between 10.0 and 20.0 in one patient (2%), and over 20.0 in three patients (7%). None of the 46 patients with the WT1cn‐change max less than 20.0 showed signs of developing MDS, such as a relapse of cytopenia and the appearance of karyotypic and/or morphological abnormalities during the follow‐up period. In contrast, two (AA26 and AA63) of the three patients whose WT1cn‐change max was above 20.0 (range 20.3–466.7) progressed to MDS (Figure 2A). These two patients showed a steady increase in WT1cn, and in one patient (AA26), the ratio decreased dramatically after allo‐HSCT. Figure 2B shows an increase in WT1cn in a patient with AA (AA63) associated with the evolution to MDS–MLD with monosomy 7. Figure 2C illustrates the changes in blood cell counts in one AA patient (AA31), in whom the WT1cn level was persistently high at approximately 600 but the WT1cn‐change max was 3.5, and no signs of developing MDS were detected.

With the exception of one case (MDS03) whose WT1cn (2800 copies) did not increase during a follow‐up period of 2 months, WT1cns of four patients with MDS were high at 1000–120,000 (median 5700) and gradually increased, with WT1cn‐change max exceeding 20.0 (23.0–190.0; median 80.0) in two patients (MDS04 and MDS05) (Figure S1A). In three patients (MDS01, MDS02, and MDS05), the ratios decreased significantly after allo‐HSCT.

In the five PNH patients whose PB samples were serially examined, the WT1cn change ratio remained stable between 1.0 and 3.1 (median 2.4) during the follow‐up period of 9–75 months (Figure S1B).

### 3.5 WT1 gene expression in granulocytes from patients with AA

**FIGURE 3:**
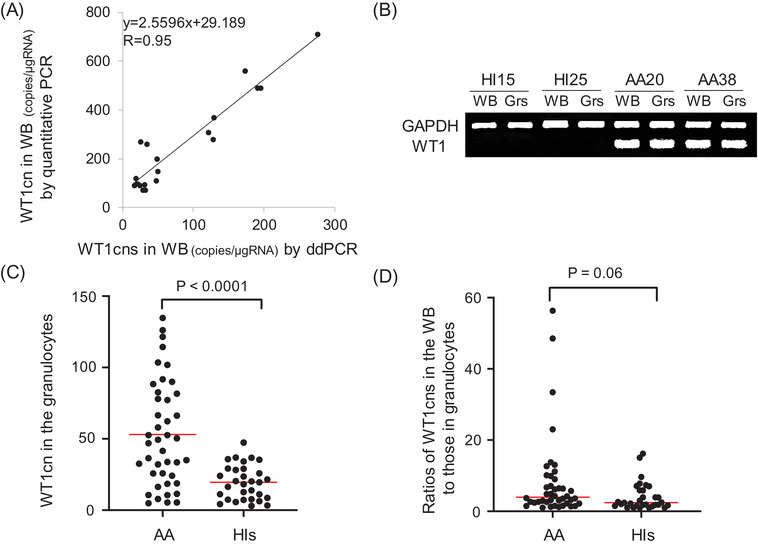
WT1 gene expression in the peripheral blood of aplastic anemia (AA) patients. (A) Correlation between the *WT1* mRNA copy numbers (WT1cns) in the whole blood (WB) detected by droplet digital polymerase chain reaction (ddPCR) and those detected by quantitative PCR in 14 patients, including 12 AA and two myelodysplastic syndromes (MDS) patients. (B) *WT1* gene expression in the WB and granulocytes of two AA patients and two healthy individuals (HIs) detected by qualitative PCR. (C) The WT1cns in the granulocytes of AA patients (*n* = 27) and HIs (*n* = 31) (*p* < 0.0001). (D) The ratios of the WT1cns in the WB to those in granulocytes of AA patients (*n* = 27) and HIs (*n* = 31) (*p* = 0.06). Abbreviation: Grs, granulocytes

Since nearly 40% of untreated cases were positive for WT1cn in association with hematologic recovery early after treatment without any signs of developing MDS, we suspected that the elevated WT1cn in PB might reflect changes in mature blood cells, such as granulocytes, rather than HSC abnormalities [20, 21]. To test this hypothesis, we developed a ddPCR method to determine WT1cn in granulocytes from patients with AA. First, we validated the accuracy of the ddPCR results by comparing the results of WT1cn measurement in whole blood (WB) with those obtained using the Otsuka kit, which was originally designed to measure WT1cn in WB. There was a strong correlation between the WT1cn values obtained with ddPCR and those obtained using quantitative PCR (*R* = 0.95) (Figure 3A). The qualitative PCR assay revealed increased WT1 gene expression in granulocytes from AA patients compared with granulocytes from HIs (Figure 3B). As shown in Figure 3C, WT1cn in isolated granulocytes from 27 AA patients was 2.5‐fold higher than that from 31 HIs. The median value of WT1cn in granulocytes converted to the unit of quantitative PCR was 48.1 (4.8–134.7) copies/μg RNA in 27 AA patients, which was significantly higher than that in 31 HIs (19.5 [3.0–47.4] copies/μg RNA, *p* < 0.0001). The median ratio of WT1cns in the WB to those in granulocytes of 27 AA patients (3.9 [1.0–56.3]) was comparable to that of 31 HIs (2.4 [1.0–16.2]) (*p* = 0.06, Figure 3D), suggesting that in addition to granulocytes, WT1 gene expression in the non‐granulocyte population of PB leukocytes is also increased in AA patients.

### 3.6 Correlation of somatic mutations with an increase in WT1cn

Deep sequencing revealed somatic mutations in genes associated with myeloid malignancies in five of 27 patients with AA, two of two with MDS, and one of three with PNH (Table 4). The presence of mutations in the five AA patients with *KIT* (AA15, variant allele fraction [VAF]; 0.448), *BCOR* and *TET2* (AA33, VAF; 0.083 and 0.0375, respectively), *BCOR* (AA58, VAF; 0.040), and one PNH patient with *SMC3* (PNH03, VAF; 0.049) was not associated with an increase in WT1cn. Although two AA patients with somatic mutations (*PPMID* tandem duplication in AA07 and ASXL1 and *PPMID* in AA46) showed high maximum WT1cns (560 and 580), their WT1cn change ratios were less than five. On the other hand, one patient with MDS who had an *EZH2* (MDS04) mutation showed increased WT1cns with a WT1cn change ratio greater than 60.0. In one patient with MDS (MDS03) who had mutations in *BCOR*, *DNMT3A*, and *PEG3*, their association with changes in WT1cn was unclear because of the short follow‐up period. Changes in the WT1cn change ratio in theses patients who possessed somatic mutations are shown in Figure S2.

**TABLE 4:** *WT1* mRNA copy numbers (WT1cns) in patients with somatic mutations of myeloid malignancy‐related genes

	Age	Diagnosis	Gender	Initial WT1cn	Maximum WT1cn	Maximum WT1cn change ratio	Mutated gene (VAF)
AA07	35	AA	Male	300	560	1.87	*PPM1D tandem duplication* (0.025)
AA15	49	AA	Female	310	310	1.00	*KIT* (0.448)
AA33	85	AA	Female	62	170	2.74	*BCOR* (0.083), *TET2* (0.0375)
AA46	51	AA	Female	140	580	4.14	*ASXL1* (0.037), *PPM1D* (0.068)
AA58	43	AA	Male	88	200	2.27	*BCOR* (0.04)
MDS03^a^	81	MDS	Male	2800	2800	1.00	*BCOR*, *DNMT3A*, *PEG3*
MDS04^a^	76	MDS	Male	2000	120,000	60.00	*EZH2*
PNH03	54	PNH	Female	77	110	1.43	*SMC3* (0.049)

Abbreviations: AA, aplastic anemia; MDS, myelodysplastic syndromes; PNH, paroxysmal nocturnal hemoglobinuria.

^a^ VAF data were not available in these two patients.

### 3.7 Survival and disease progression during the clinical course

**FIGURE 4:**
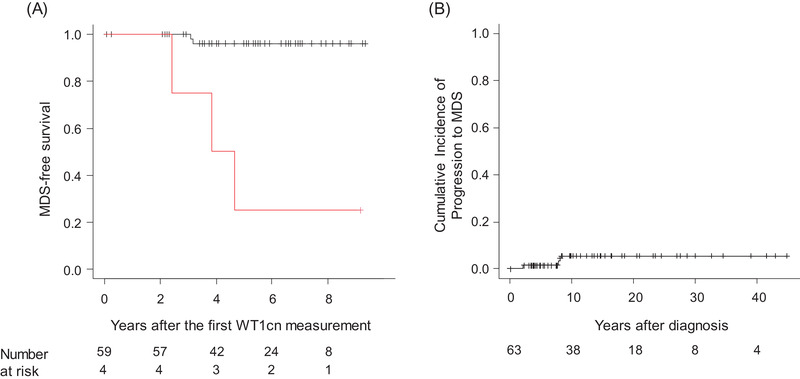
Survivals and disease progression in aplastic anemia (AA) patients. (A) Probability of myelodysplastic syndromes (MDS)‐free survival of four patients whose the *WT1* mRNA copy number (WT1cn)‐change max >20 (red) and 59 patients with the WT1cn‐change max <20 (black). (B) Cumulative incidence of disease progression to MDS.

The 10‐ and 20‐year overall survival rates of the 63 AA patients from the time of the first WT1cn measurement were 97.7% and 90.3%, respectively (95% confidence interval [CI]: 84.6%–99.7% and 72.1%–96.9%, respectively). The MDS‐free survival of 59 AA patients with a WT1cn‐change max <20 at 5 years (96.0%) was higher than that of four AA patients with a WT1cn‐change max ≥20 (25.0%) (Figure 4A). Three patients died during follow‐up due to HSCT‐related toxicity (AA14), fatal arrhythmia due to hemochromatosis (AA26), and liver abscess (AA62). The cumulative incidence of progression to MDS was 6.2% (95% CI: 2.0–18.2; Figure 4B) at 10 years.

## 4 DISCUSSION

This study revealed that WT1cn, a reliable marker of leukemic burden, was moderately elevated in approximately 40% of patients with untreated AA, and 70% of patients who were negative for WT1cn before treatment became positive 3–78 months after treatment, with no evidence of developing secondary MDS/AML, although WT1cn levels dropped repeatedly in some patients and rose again after they became positive. These results contrast with those of previous studies that failed to detect an increase in WT1cn in any AA patient [16]. Given that the WT1 gene expression was enriched in granulocytes in AA patients, the discrepancy could be explained by the lower neutrophil count of AA patients in the previous study (*n* = 6; median 227; range 56–820) than in our patients (median 1320; range 60–2952) [16]. WT1cn reflects the proliferative potential of cancer cells or HSCs [22, 23, 24]; however, several studies have shown that *WT1* gene expression is detectable in mature granulocytes [20, 21]. In line with this report, WT1cns in granulocytes from AA patients whose PB showed increased WT1cns were apparently higher than those of HIs.

The moderate increase in WT1cn in AA patients can confuse the physicians in charge of their treatment because such an increase in WT1cn levels has been demonstrated in patients with low‐risk MDS [4, 25]. Despite the persistence of moderately high WT1cns, most AA patients remained in stable remission without apparent signs of clonal evolution. Therefore, it is important to bear in mind that the increase in WT1cn up to 600 copies or in the WT1cn change ratio up to 20.0 does not necessarily represent the transformation of AA to MDS, which may necessitate therapy with hypomethylating agents and allo‐HSCT. In contrast, a steady increase in WT1cn resulting in a high (>20) WT1cn change ratio was predictive of secondary MDS in our study cohort. Serial measurements of WT1cn at regular intervals may thus be useful for diagnosing the transformation of AA to MDS.

Recent studies have revealed that somatic mutations are detected in approximately 30% of AA patients, and mutations in several genes such as *DNMT3A*, *ASXL1*, and *RUNX1* are associated with secondary myeloid malignancies [14, 26]. We reasoned that the increased WT1cns in some AA patients could be explained by the presence of somatic mutations. However, there was no correlation between the presence of somatic mutations including *TET2* and *ASXL1* and an increase in the WT1cn change ratio of our AA and PNH patients. The presence of somatic mutations at low VAFs may be irrelevant to the clonal evolution of MDS/AML, particularly when they are not associated with increased WT1cn levels. The lack of predictive value for somatic mutations in the absence of increased WT1cn levels needs to be confirmed by a larger number of patients.

The reason for the moderate increase in WT1 gene expression in granulocytes from AA patients during convalescence after treatment remains obscure. Our recent study showed that immune attack on HSC persists even in AA patients who achieve complete response after successful IST and favors the proliferation of human leukocyte antigens class I allele‐lacking HSCs [27]. Inflammatory cytokines secreted during persistent immune attack may prevent HSCs from undergoing effective maturation, which eventually leads to increased WT1 gene expression in their progeny. Signs of ineffective hematopoiesis, such as high mean corpuscular volumes and a slight increase in lactate dehydrogenase levels, are often observed in convalescent patients with AA [28, 29]. An increased WT1 gene expression has been demonstrated in mature granulocytes in the BM of patients with MDS [30, 31]. Therefore, the WT1cn increase in patients after IST may reflect ineffective hematopoiesis rather than the presence of abnormal HSC clones.

The limitation of this study is that the observation period of AA patients (median 46 months) may not have been long enough to undergo clonal evolution. Although none of our AA patients with a WT1cn change ratio as high as 20.0 developed MDS/AML, some patients with moderately high WT1cn could be at potential risk for clonal evolution after a longer observation period. Further observation is needed to clarify the significance of elevated WT1cn in AA patients in remission. Also, the incidence of secondary MDS in this study cohort (6.2% at 10 years) was lower than that previously reported by other researchers. Groarke et al. [30] reported that the incidence of clonal evolution in non‐severe AA patients is lower than that in SAA patients. The high proportion of patients with non‐severe AA among the untreated (77%) and treated (89%) patients may be a reason for the relatively lower incidence of MDS in this study.

In conclusion, a moderate increase in WT1cns up to several hundred is common in AA patients in remission and is attributable to increased WT1 gene expression by mature granulocytes. Patients with a WT1cn change ratio greater than 20.0 may be at increased risk of developing MDS and need careful monitoring.

## AUTHOR CONTRIBUTIONS

Ken Ishiyama, Tran Cao Dung, and Shinji Nakao designed the research, collected clinical data, and wrote the manuscript. Ken Ishiyama and Tran Cao Dung organized the project and analyzed the data. Tran Cao Dung and Kohei Hosokawa performed flow cytometry, and qualitative and droplet digital PCR. Tatsuya Imi and Hirohito Yamazaki managed patients and prepared samples. Yasuhito Nannya and Seishi Ogawa performed deep sequencing. All authors critically reviewed the manuscript and checked the final manuscript.

## CONFLICT OF INTEREST

The authors declare they have no conflicts of interest.

## ETHICS STATEMENT

This study was approved by the Institutional Review Board (2468).

## PATIENT CONSENT STATEMENT

All participants gave their informed written consent to participate in this study.

## Supporting information


**Data S1**. Therapies given to the untreated aplastic anemia (AA) patients
**Data S2**. Detection of somatic gene mutations
**Data S3**. Measurement of *WT1* mRNA copy number (WT1cn) in granulocytes using droplet digital polymerase chain reaction (ddPCR)
**Data S4**. Determination of the *WT1* mRNA copy number (WT1cn) in granulocytes
**Data S5**. Qualitative polymerase chain reaction (PCR) to detect WT1 gene expression in peripheral blood (PB) and granulocytes
**Data S6**. Statistical methods
**FIGURE S1**. Changes in the *WT1* mRNA copy number (WT1cn) change ratio over time in patients with myelodysplastic syndrome (MDS) and paroxysmal nocturnal hemoglobinuria (PNH).
**FIGURE S2**. Changes in the *WT1* mRNA copy number (WT1cn) change ratio over time in patients who possessed somatic gene mutations associated with myeloid malignancies.

## Data Availability

The data that support the findings of this study are available from the corresponding author upon reasonable request.
