# Supplementary material for: Clinical significance of the increased expression of the WT1 gene in peripheral blood of patients with acquired aplastic anemia
Source: EJHaem. 2022 Sep 20;3(4):1116–25. doi: 10.1002/jha2.563 (PMC9713059; doi:10.1002/jha2.563)
Supplement: Supplementary file 1 — Data S1. Therapies given to the untreated aplastic anemia (AA) patients Data S2. Detection of somatic gene mutations Data S3. Measurement of WT1 mRNA copy number (WT1cn) in granulocytes using droplet digital polymerase chain reaction (ddPCR) Data S4. Determination of the WT1 mRNA copy number (WT1cn) in granulocytes Data S5. Qualitative polymerase chain reaction (PCR) to detect WT1 gene expression in peripheral blood (PB) and granulocytes Data S6. Statistical methods FIGURE S1. Changes in the WT1 mRNA copy number (WT1cn) change ratio over time in patients with myelodysplastic syndrome (MDS) and paroxysmal nocturnal hemoglobinuria (PNH). FIGURE S2. Changes in the WT1 mRNA copy number (WT1cn) change ratio over time in patients who possessed somatic gene mutations associated with myeloid malignancies. [file JHA2-3-1116-s001.docx]

**Supportive information**

**Clinical significance of the increased expression of the WT1 gene in peripheral blood of patients with acquired** **aplastic anemia**

Ken Ishiyama^1*^, Tran Cao Dung^1*^, Tatsuya Imi^1^, Kohei Hosokawa^1^, Yasuhito Nannya^2,3^, Hirohito Yamazaki^1^, Seishi Ogawa^2,4,5^ and Shinji Nakao^1^.

1: Department of Hematology, Kanazawa University Hospital, Kanazawa, Ishikawa, Japan.

2: Department of Pathology and Tumor Biology, Graduate School of Medicine, Kyoto University, Kyoto, Japan.

3: Division of Hematopoietic Disease Control, Institute of Medical Science, The University of Tokyo, Tokyo, Japan.

4: Institute for the Advanced Study of Human Biology, Kyoto University, Kyoto, Japan.

5: Center for Hematology and Regenerative Medicine, Karolinska Institutet, Stockholm, Sweden.

*These authors contributed equally to this work.

Correspondence: Shinji Nakao, M.D., Ph.D.

Department of Hematology, Kanazawa University Hospital, 13-1 Takaramachi, Kanazawa, Ishikawa, 920-8641, Japan.

E-mail: snakao8205@staff.kanazawa-u.ac.jp

Phone: +81-76-265-2275, FAX: +81-76-234-4252

**Supporting Information**

**Data S1.** Therapies given to the untreated aplastic anemia (AA) patients

**Data S2.** Detection of somatic gene mutations

**Data S3.** Measurement of *WT1* mRNA copy number (WT1cn) in granulocytes using droplet digital PCR (ddPCR)

**Data S4.** Determination of the WT1cn in granulocytes

**Data S5.** Qualitative PCR to detect WT1 gene expression in the whole blood (WB) and granulocytes

**Data S6.** Statistical methods

**Figure S1.** Changes in the WT1cn change ratio over time in patients with myelodysplastic syndrome (MDS) and paroxysmal nocturnal hemoglobinuria (PNH)

**Figure S2.** Changes in the WT1cn change ratio over time in patients who possessed somatic gene mutations associated with myeloid malignancies (n=8)

**Data S1.** **Therapies given to the untreated AA patients**

The patients were treated with rabbit antithymocyte globulin (ATG) plus cyclosporine (CsA) in six, CsA alone in seven, thrombopoietin receptor agonists (TPO-RA) in 15 (data were missing in one patient) after blood collection. Of the 13 patients treated with immunosuppressive therapy, six achieved a complete response (CR) and seven a partial response (PR). Two of the three nonresponders underwent allo-HSCT. One CR (AA27) and one PR (AA28) patient later developed myelodysplastic syndrome (MDS) and hemolytic paroxysmal nocturnal hemoglobinuria (PNH), respectively.

**Data S2. Detection of somatic gene mutations**

Deep sequencing was performed at Kyoto University to search for gene abnormalities associated with myeloid malignancies in 32 patients with an increase in changes in WT1cn (1, 2). Three hundred and seventy-seven previously mutated genes in patients with BM failure were selected for targeted capture with the SeqCap EZ choice (Roche Diagnostics, Westfield, IN, USA). All DNA libraries were prepared according to the manufacturer's instructions. Captured targets were subjected to paired-end sequencing using the Illumina MiSeq System (San Diego, CA, USA). Mutations with >20% variant allele frequency (VAF) were validated by Sanger sequencing, whereas mutations with <20% VAF were validated by resequencing with MiSeq.

**Data S3. Measurement of WT1cn in granulocytes using ddPCR**

The sediment obtained after centrifugation of diluted PB on Lymphoprep™ (Axis-Shield PoC AS, Oslo, Norway, #1114544) was lysed with ammonium chloride buffer. Mature granulocytes were obtained after washing the lysates with phosphate-buffered saline. The percentage of granulocytes to total leukocytes in the pellet was 87.6%­–92.5%. A total of 1.2–2.0 μg of RNA extracted from the granulocytes was subjected to one-step duplex ddPCR (3, 4). Supermix, reverse transcriptase, and dithiothreitol were included in the One-Step RT-ddPCR Advanced Kit for Probes (Bio-Rad Laboratories Inc., #1864021). Primers and probes with dye-labeled FAM-minor groove binder (MGB). The thermal cycling was performed in a C1000 Touch^TM^ Thermal Cycler of QX200 ddPCR system (Bio-Rad) using a ramp rate of 2^o^C per second and the following protocol: 50^o^C for 60 minutes for reverse transcription, 95^o^C for 10 minutes for enzyme activation, followed by 45 cycles of denaturation at 95^o^C for 30 seconds, and annealing and extension at 60^o^C for 90 seconds, finishing with one cycle at 98^o^C for 10 minutes for enzyme deactivation and holding at 4^o^C infinitely. ddPCR results were deemed reliable only when the copy number of *ABL1* mRNA in 20 μL of the reaction mixture was greater than 10,000 copies (5).

**Data S4. Determination of the WT1cn in granulocytes**

The WT1cn in granulocytes was calculated using the following formula:


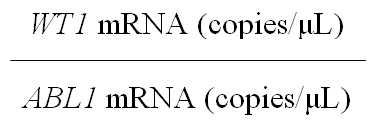


WT1cn in granulocytes (copies/μg RNA) = × 30,093.282 (copies/μg RNA)*

*30,093.282 (copies/μg RNA): the mean copy number of *ABL1* mRNA per 1 μg RNA in mature granulocytes in the PB determined in 31 healthy individuals.

**Data S5. Qualitative PCR to detect WT1 gene expression in the WB and granulocytes**

An equal amount of 2 μg RNA extracted from the WB and granulocytes was converted to cDNA using the High-Capacity cDNA Reverse Transcription Kit with RNase Inhibitor (Thermo Fisher Scientific, #4374966). The PCR reaction was performed using the HotStarTaq Plus Master Mix Kit from Qiagen (#203645), *WT1* primer as described in Data S3, *GAPDH* primer (Thermo Fisher Scientific, #402869), 2 μL cDNA, and RNase-free water in a 20 μL reaction mixture. Thermal cycling consisted of an initial 4-minute hold at 94°C, followed by a 25-second hold at 94°C, a 20-second hold at 60°C, and a 25-second hold at 72°C for 45 cycles and a final extension of 1-minute at 72°C. PCR products were electrophoretically separated on a 2% agarose gel to confirm a band of approximately 226 base pairs (bp) for *GAPDH* and a band of 72 bp for *WT1*.

**Data S6. Statistical methods**

Laboratory parameters were divided into two groups using the median for continuous variables and compared using the Mann-Whitney U test. Statistical significance was set at P <0.05. Overall survival (OS) was defined as the number of days fromthe first WT1cn measurement to last follow-up or death from any cause. The OS rate was calculated using the Kaplan-Meier method. All P values were two-sided. All statistical analyses were performed using the EZR software package (Saitama Medical Center, Jichi Medical University, Saitama, Japan) (6).

**Figure S1. Changes in the WT1cn change ratio over time in patients with MDS and PNH.** The WT1cn change ratios of individual patients are shown in different colors. a. Patients with MDS (n=5). The red arrows indicate the times of allo-HSCT. b. Patients with PNH (n=5).


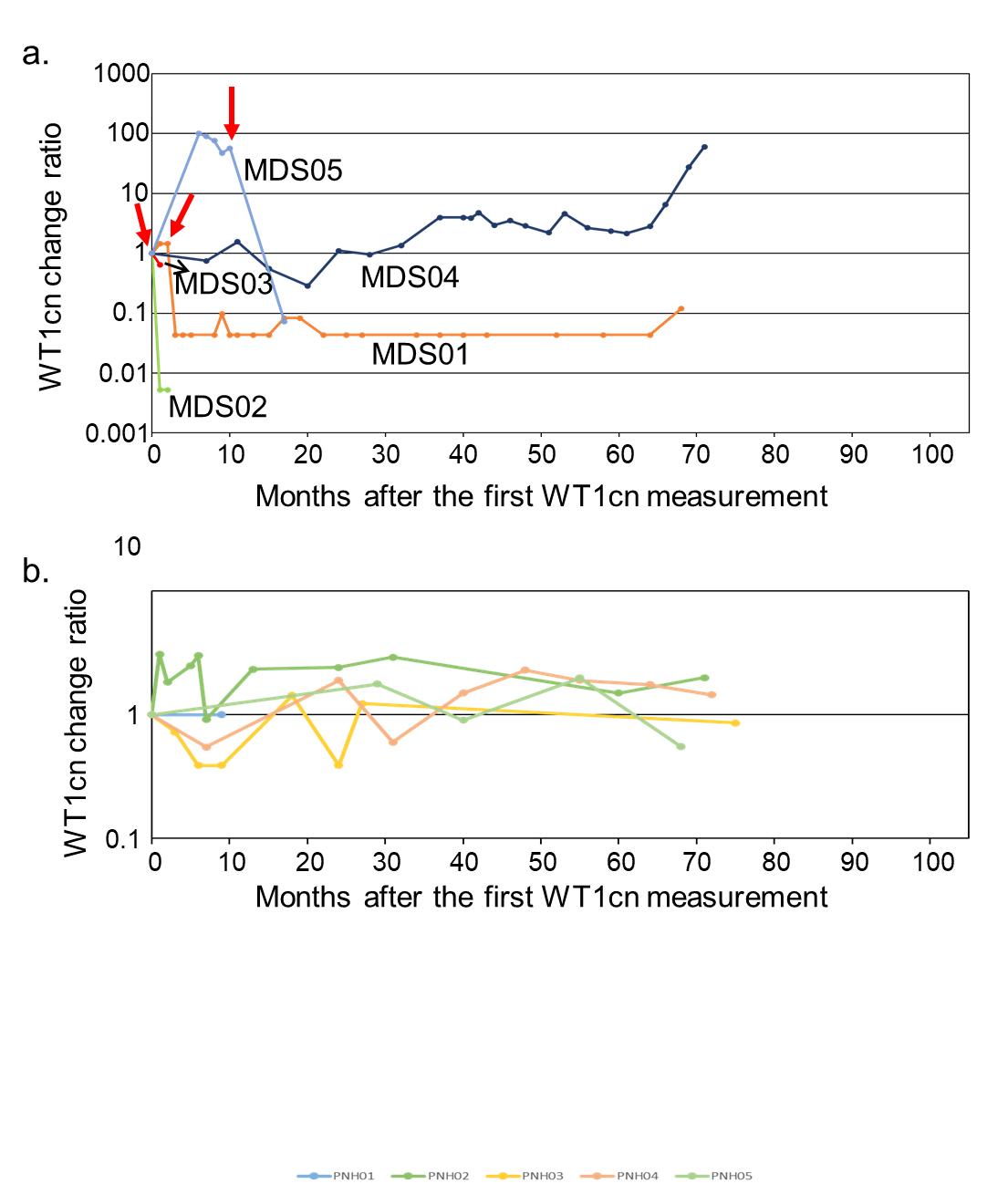


**Figure S2. Changes in the WT1cn change ratio over time in patients who possessed somatic gene mutations associated with myeloid malignancies (n=8)**


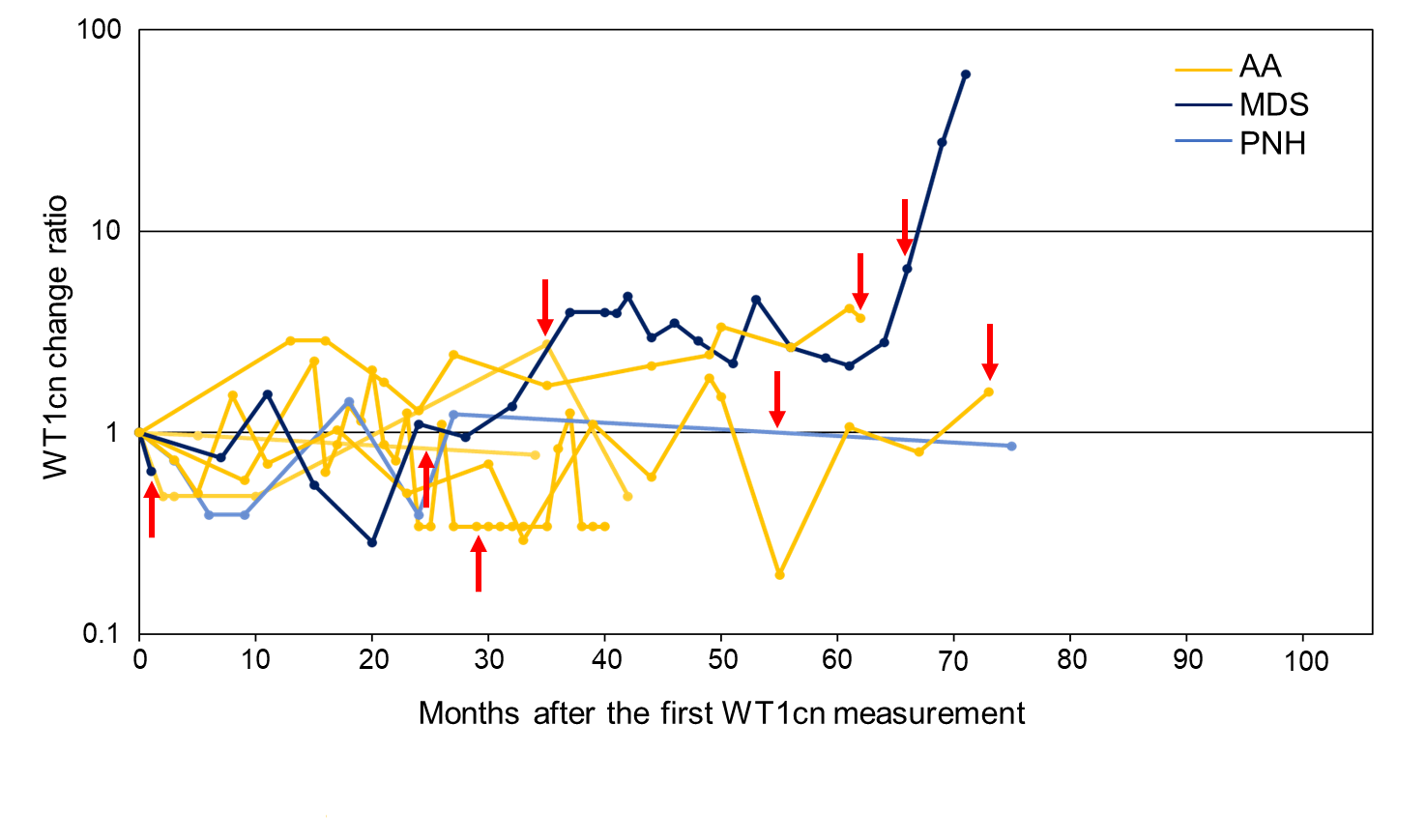


The yellow, dark blue, and light blue lines indicate that patients with AA, MDS, and PNH, respectively. Red arrows indicate the time points when deep sequencing was performed.

**References**

1. Exeter DJ, Moss L, Zhao J, Kyle C, Riddell T, Jackson R, et al. The distribution and frequency of blood lipid testing by sociodemographic status among adults in Auckland, New Zealand. J Prim Health Care. 2015;7(3):182-91.

2. Yoshizato T, Dumitriu B, Hosokawa K, Makishima H, Yoshida K, Townsley D, et al. Somatic Mutations and Clonal Hematopoiesis in Aplastic Anemia. N Engl J Med. 2015;373(1):35-47.

3. Dewispelaere L, Bleret L, Van Acker T, Van Branteghem C, Cochaux P, Heimann P, et al. One-Step Duplex Droplet Digital PCR for WT1 Overexpression. J Mol Diagn. 2020;22(8):1008-19.

4. Koizumi Y, Furuya D, Endo T, Asanuma K, Yanagihara N, Takahashi S. Quantification of Wilms' tumor 1 mRNA by digital polymerase chain reaction. Int J Hematol. 2018;107(2):230-4.

5. d MG, Huggett JF. The Digital MIQE Guidelines Update: Minimum Information for Publication of Quantitative Digital PCR Experiments for 2020. Clin Chem. 2020;66(8):1012-29.

6. Kanda Y. Investigation of the freely available easy-to-use software 'EZR' for medical statistics. Bone Marrow Transplant. 2013;48(3):452-8.
